# Correction: From gym to joy: The serial mediation of motor competence and health literacy in Chinese university students’ exercise-life satisfaction pathway

**DOI:** 10.1371/journal.pone.0343052

**Published:** 2026-02-13

**Authors:** Shao-Shuai Ma, Zhe Zhu, Dongsheng Cai, Chen-Xi Li, Ya-Xing Li, Bo Li, Sai Zhu, Jiaxian Geng

The authors, Zhe Zhu, Dongsheng Cai and Chen-Xi Li, should be noted as shared first co-authors on this work.
